# Live birth rate following frozen-thawed blastocyst transfer is higher in high-grade day 6 blastocysts than in low-grade day 5 blastocysts

**DOI:** 10.3389/fendo.2022.1066757

**Published:** 2023-01-04

**Authors:** Wenhao Shi, Hanying Zhou, Lijuan Chen, Xia Xue, Juanzi Shi

**Affiliations:** The Assisted Reproduction Center, Northwest women’s and Children’s Hospital, Xi’an, China

**Keywords:** blastocyst, day 5 vs day 6, frozen blastocyst transfer, live birth rate, blastocyst quality

## Abstract

**Background:**

Day 5 (D5) blastocysts are generally given priority to transfer than day 6 (D6) blastocysts; however, which one should be prioritized to transfer when only low-grade D5 and high-grade D6 blastocysts are available?

**Methods:**

A large retrospective cohort study was carried out to evaluate the live birth rate (LBR) following D5 and D6 blastocysts in single frozen-thawed blastocyst transfer (FBT) during January 2014 and December 2018. A multivariate logistic regression was conducted to evaluate the combined impact of expansion day (D5 and D6) and blastocyst quality (high grade/low grade) on LBR, accounting for the potential confounding factors. The biopsied blastocysts from a consecutive PGT-A case series during February 2013 to December 2021 were analyzed in a supplementary study.

**Results:**

The LBR achieved in high-grade D6 blastocyst transfer was significantly higher than that in low-grade D5 blastocyst transfer (50.43% vs. 40.70%, aOR 1.54, 95% CI 1.05–2.26, *p* = 0.027). There were no significant differences in preterm birth rate, very preterm birth rate, mean live birth weight, and birth weight <1,500 g and >4,000 g between the two cohorts. As for aneuploidy analysis in PGT, there were 54.55% of euploid blastocysts (30/55) among high-grade D6 blastocysts, significantly higher than the 41.39% of euploid blastocysts (565/1,365) among low-grade D5 blastocysts (*p* < 0.001).

**Conclusions:**

Our data suggest that D6 blastocysts with high morphology grading are preferred than D5 blastocysts with low morphology grading when selecting blastocyst transfer to shorten the time of conception.

## Introduction

Embryos cultured *in vitro* typically develop to expanded blastocysts on day 5 (D5) for transfer or cryopreservation. Some slower blastocysts that failed to expand on D5 were prolonged a day later [day 6 (D6)] until their expansion and then preserved to increase the chance of pregnancy per IVF cycle (1), accounting for 12.19% of valid blastocysts in our clinic. Studies have indicated that D5 blastocysts have a higher chance of being euploid (2) and have a higher clinical pregnancy and live birth rate (LBR) than D6 blastocysts (3, 4). Hence, in general clinical practice, the D5 blastocyst is given priority to transfer than the D6 blastocyst when selecting embryos to transfer for frozen-thawed blastocyst transfer (FBT) (5).

However, in cases where only low-grade D5 and high-grade D6 blastocysts are available, it is difficult to decide which one should be transferred first. According to the policy above, it appears reasonable to transfer D5 poor-quality blastocysts first. Paradoxically, a high-grade blastocyst is preferred since quality specific to the morphology grading is also critical to the pregnancy outcome (6, 7). Owing to insufficient data on the combined impact of expansion day (D5 and D6) and blastocyst quality (high grade/low grade) on live birth, it would be difficult for the embryologist to decide which between low-grade D5 and high-grade D6 blastocysts should be prioritized to transfer. Optimal selection of embryos can help to minimize the time to pregnancy during an *in vitro* fertilization embryo transfer IVF-ET treatment. If it is inconsistent to achieve a better outcome after a D5 poor blastocyst transfer compared to a D6 excellent blastocyst transfer, it could potentially increase time to live birth for IVF patients. Thus, a retrospective cohort study was conducted to investigate LBR following frozen-thawed embryo transfer resulting from low-grade D5 vs. high-grade D6 blastocysts.

## Methods

### Study design

This is a retrospective cohort study that included all single FBT between January 2014 and December 2018 in a public outpatient clinic. The follow-up date was until 31 December 2019. The deidentified data were extracted from the electronic medical record system (Wuhan Huchuang, Co., Ltd.; version 9.2.5.8). The pregnancy and perinatal outcomes were compared between four groups of blastocysts: (i) high-grade D5 blastocysts; (ii) low-grade D5 blastocysts; (iii) high-grade D6 blastocysts; and (iv) low-grade D6 blastocysts. The biopsied blastocysts from a consecutive PGT-A case series during February 2013 to December 2021 were analyzed as a supplementary study to access the chromosomal abnormalities between four groups.

### Participants

A total of 4,473 FBT cycles with a single-embryo transfer were included during the study period. The following cycles were excluded: (i) day 7 (D7; *n* = 10); (ii) patients who have uterine infertility as intrauterine adhesion, submucosal fibroids or polyps, intramural fibroids > 4 cm, and congenital uterine malformation (*n* = 75); (iii) patients who have hypertension, diabetes, or thyroid dysfunction (*n* = 33); and (iv) unexpanded blastocysts and missing data of blastocyst grading or live birth (n = 159). Four cohorts were compared according to the day and the quality of blastocyst: (i) high-grade D5 blastocysts; (ii) low-grade D5 blastocysts; (iii) high-grade D6 blastocysts; and (iv) low-grade D6 blastocysts.

### IVF procedure

For ovarian stimulation, patients were treated with recombinant and/or urinary gonadotrophins in a long gonadotropin-releasing hormone (GnRH) agonist or a GnRH antagonist protocol. Embryos were cultured individually in 50 μl of sequential medium droplets (Vitrolife, Sweden) after conventional IVF or ICSI fertilization. The detailed procedures were described in our previous studies (8, 9). For the cases with sufficient high-quality embryos (more than two to four) on day 3, extended blastocyst culture was practiced. Blastocyst transfer was carried out on D5 in fresh cycles. The surplus blastocysts were cryopreserved on D5 or D6. For a blastocyst to be frozen, it should be fully expanded. Vitrification was performed as the method of cryopreservation using Cryotop, an open system (Kitazato BioPharmaCo, Japan). The vitrification and warming procedure were conducted according to standard protocols, as previously described (10, 11). The frozen blastocysts were warmed and cultured for 2–3 h before they are transferred on the day of embryo transfer.

### FBT protocol

The endometrial preparation for FBT was performed according to standard protocol in either a natural or an artificial cycle (hormone treatment protocol). In a natural cycle, ovarian follicle was monitored as well as serum hormone to confirm the day that a spontaneous ovulation occurred or triggered by 10,000 IU of human chorionic gonadotropin. The day of blastocyst transfer was usually programed on the 6th day of ovulation. In an artificial cycle, oral estradiol valerate (Progynova; Bayer Schering Pharma AG) was administered at a daily dose of 6 mg from D5 of menstrual cycle. When the thickness of the endometrium was at least 7–8 mm based on transvaginal ultrasound, 60 mg/day of progesterone was started for luteal phase support until a negative serum β-hCG was obtained or until 8 weeks of pregnancy. The day of blastocyst transfer was usually programed on the 7th day after the start of progesterone treatment.

### Blastocyst morphology

Blastocyst morphology was assessed on D5 (twice, 118 ± 1 h and 124 ± 1 h post-insemination) and D6 (142 ± 1 h post-insemination) based on the Gardner grading system (12). Only blastocysts that achieved the expanded stage were vitrified and included in this study. Inner cell mass (ICM) and trophectoderm (TE) were classified according to the number and structure of cells. The appearance of ICM/TE with many cells tightly packed was defined as “A”; the appearance of ICM/TE with several grouped cells was defined as “B”; the appearance of ICM/TE with a few loose cells was defined as “C”. The high-grade blastocyst was considered as an expanded, hatching, or hatched blastocyst with high ICM/TE grading (AA, AB, BA, and BB), and other classifications (AC, CA, BC, CB, and CC) were considered as low ICM/TE grading in this study. The choice of the blastocyst to transfer was based primarily on the day of expansion and secondly on the Gardner scoring system: high-grade D5 blastocysts > low-grade D5 blastocysts > high-grade D6 blastocysts > low-grade D6 blastocysts.

### Outcome measurements

The primary outcome measurement was LBR, in which the live birth was defined as the delivery of any viable infant at 28 weeks of gestation or longer after the blastocyst transfer. Secondary outcomes included clinical pregnancy rate, preterm birth rate, and neonatal outcomes. Biochemical pregnancy was assessed by serum β-hCG level 12 days after blastocyst transfer. Clinical pregnancy was defined as the presence of intrauterine gestational sac, with or without fetal heartbeat on ultrasonography in the first trimester. Preterm birth is defined as a live birth or stillbirth that takes place after at least 28 but before 37 completed weeks of gestational age. The neonatal outcomes included sex ratio and birth weight.

### Chromosome aneuploidy analysis

The biopsied blastocysts from a consecutive PGT-A case series during February 2013 to December 2021 were performed as a supplementary analysis to access the euploidy rate between high-grade D5 blastocysts, low-grade D5 blastocysts, high-grade D6 blastocysts, and low-grade D6 blastocysts. Blastocysts that presented with a good- or fair-quality inner cell mass and trophectoderm were biopsied and screened for aneuploidy utilizing a targeted next-generation sequencing (NGS). Mosaic blastocysts were included in aneuploid embryos for statistics.

### Statistical analysis

All data management and analyses were conducted by EmpowerStats software (www.empowerstats.com version R.3.4.3). The continuous variables were presented as median (Q1−Q3), whereas categorical variables were presented as counts and proportions. The differences were evaluated by the Kruskal–Wallis test for continuous variables and chi-square test. A multivariate logistic regression was conducted to evaluate the combined impact of expansion day (D5/D6) and blastocyst quality (high grade/low grade) on IVF outcome (live birth, yes/no), accounting for the following potential confounding factors: female age (smooth), parity (0, 1, and ≥2), ET times (1st, 2nd, and ≥3rd), and blastocyst status (expanded, hatching, and hatched). A multivariable logistic model was constructed, which accounted for the blastocyst quality (high grade vs. low grade) and blastocyst day (D5 vs. D6) within each FBT. Results are presented as adjusted odds ratios (ORs) with 95% confidence intervals (CIs). *p* < 0.05 was considered as statistically significant.

## Results

### Baseline characteristics

An initial cohort of 4,473 blastocysts in a single FBT were used in this study period. After the standard screening (excluding blastocysts from D7, patients who have uterine infertility, patients who have hypertension, diabetes, or thyroid dysfunction, and missing data), the remaining 4,202 valid blastocysts that originated from D5 or D6 were used for further analysis. Four groups were compared according to expansion day and the quality of blastocyst: (i) high-grade D5 blastocysts, *n* = 1835; (ii) low-grade D5 blastocysts, *n* = 1791; (iii) high-grade D6 blastocysts, *n* = 115; and (iv) low-grade D6 blastocysts, *n* = 461.

There were 576 D6 blastocysts, accounting for 13.71% of single-blastocyst transfers (vs. 86.29% of D5 blastocysts). Of these D6 blastocysts, 115 blastocysts (19.97%) were identified as high-grade blastocysts and 461 were identified as low-grade blastocysts (80.03%). There were 1,835 high-grade D5 blastocysts (50.61%) and 1,791 low-grade blastocysts (49.39%). The baseline characteristics of two main groups (low-grade D5 vs. high-grade D6) are presented in **Table 1** and the other groups are presented in **Table S1**. The basic parameters including female age, antral follicle count, body mass index, etiology of infertility, type of infertility, gravidity, parity, FBT protocol, and endometrial thickness were comparable between the two main groups. A higher percentage of first-time embryo transfer (35.51% vs. 24.35%, *p* = 0.011) and expanded blastocyst (97.10% vs. 92.17%, *p* = 0.004) was determined in low-grade D5 blastocysts compared with high-grade D6 blastocysts (**Table 1**).

**Table 1:** Patient and cycle characteristics according to blastocyst day and morphology grading.

Blastocyst	Low-grade Day 5	High-grade Day 6	*p*-value
*N*	1,791	115	
Female age at OPU	30.00 (28.00–33.00)	30.00 (28.25–33.00)	0.720
Female age at ET	31.00 (28.00–34.00)	31.00 (29.00–34.00)	0.694
Female age at ET			0.724
<35 years	1,411 (78.78%)	89 (77.39%)	
≥35 years	380 (21.22%)	26 (22.61%)	
BMI	22.02 (20.02–24.40)	22.00 (19.83–23.92)	0.792
AFC	12.00 (9.00–16.75)	12.00 (9.00–16.00)	0.643
Etiology of infertility			0.374
Pelvic-tubal factor	887 (49.53%)	66 (57.39%)	
PCOS	224 (12.51%)	14 (12.17%)	
Male factor	488 (27.25%)	24 (20.87%)	
Other reasons	224 (12.51%)	14 (12.17%)	
Type of infertility			0.628
Primary	677 (38.42%)	46 (40.71%)	
Secondary	1,085 (61.58%)	67 (59.29%)	
Gravidity			0.966
0	698 (38.97%)	46 (40.00%)	
1	513 (28.64%)	33 (28.70%)	
≥2	580 (32.38%)	36 (31.30%)	
Parity			0.943
0	1,254 (70.02%)	82 (71.30%)	
1	500 (27.92%)	31 (26.96%)	
≥2	37 (2.07%)	2 (1.74%)	
ET times			**0.011**
1st	636 (35.51%)	28 (24.35%)	
2nd	944 (52.71%)	65 (56.52%)	
≥3rd	211 (11.78%)	22 (19.13%)	
Blastocyst status			
Expanded	1,739 (97.10%)	106 (92.17%)	**0.004**
Hatching	38 (2.12%)	5 (4.35%)	
Hatched	14 (0.78%)	4 (3.48%)	
FBT protocol			0.987
Natural cycle	217 (12.12%)	14 (12.17%)	
Artificial cycle	1573 (87.88%)	101 (87.83%)	
Endometrial thickness	10.00 (9.00–11.20)	10.55 (9.03–11.97)	0.389

AFC, antral follicle count; BMI, body mass index; ET, embryo transfer; FBT, frozen-thawed blastocyst transfer; OPU, oocyte pick-up; PCOS, polycystic ovary syndrome.

Median (Q1–Q3)/N (%).

Kruskal–Wallis test for continuous variables.

Chi-square for categorical variables.

Statistically significant values are highlighted in bold.

### Perinatal outcomes

**Figure 1:**
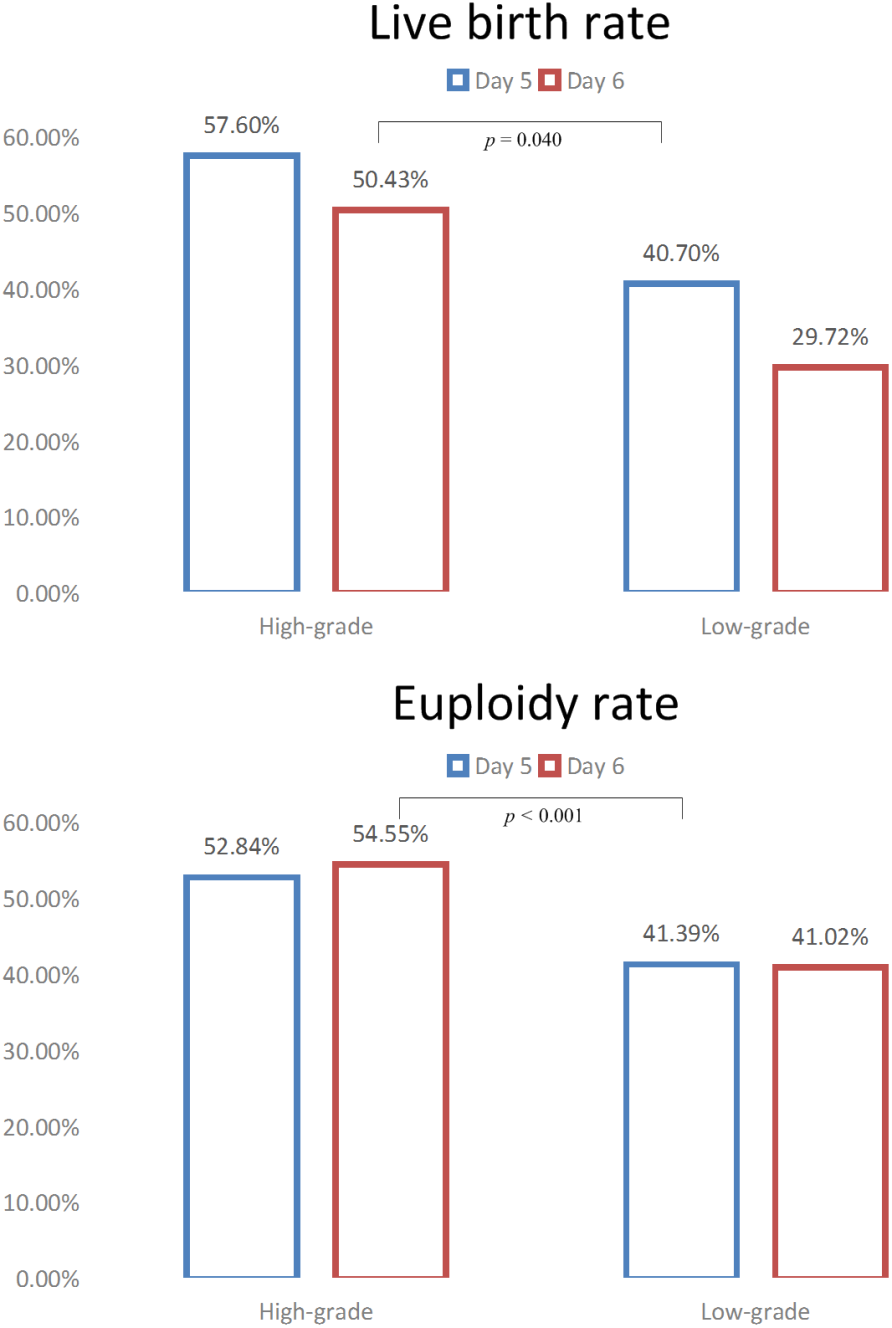
The live birth rates and euploidy rates according to blastocyst grading and expansion day.

The clinical pregnancy rate and LBR were highest in high-grade D5 blastocysts (69.43% and 57.60%), followed by high-grade D6 blastocysts (62.61% and 50.43%) and low-grade D5 blastocysts (53.10% and 40.70%), and were lowest in low-grade D6 blastocysts (39.26% and 29.72%) (**Table 2** and **Table S2**). The clinical pregnancy rate and LBR were significantly higher in high-grade D6 blastocysts than in low-grade D5 blastocysts (*p* = 0.047 and *p* = 0.040) (**Figure 1**). However, the preterm birth rate was comparable between these groups as well as very preterm birth rate, birth weight of newborns, and sex ratio.

**Table 2:** Perinatal outcomes according to blastocyst day and morphology grading.

Blastocyst	Low-grade Day 5	High-grade Day 6	*p*-value
Biochemical pregnancy	1,052 (58.74%)	78 (67.83%)	0.054
Clinical pregnancy	951 (53.10%)	72 (62.61%)	**0.047**
Live birth	729 (40.70%)	58 (50.43%)	**0.040**
Preterm deliveries (<37 weeks)	82 (11.25%)	8 (13.79%)	0.558
Very preterm deliveries (<32 weeks)	9 (1.23%)	0 (0.00%)	0.395
Birth weight	3,400.00 (3,050.00–3,700.00)	3,375.00 (3,025.00–3,700.00)	0.806
<1,500 g	11/740 (1.49%)	0/58	-
<2,500 g	52/740 (7.03%)	1/58 (1.72%)	-
>4,000 g	70/740 (9.46%)	3/58 (5.17%)	-
Birth weight in singlet pregnancy	3,400.00 (3,100.00–3,700.00)	3,375.00 (3,025.00–3,700.00)	0.907
Sex ratio (female/male)	362/378	28/30	0.924

Median (Q1–Q3)/N (%).

Kruskal–Wallis test for continuous variables.

Chi-square for categorical variables.

Statistically significant values are highlighted in bold.

### Multivariate analysis

A multivariate logistic regression analysis was performed (**Table 3**) to calculate the ORs between low-grade D5 and high-grade D6 blastocysts adjusted for female age (≤35 and >35 years), parity (0, 1, and ≥2), ET times (1st, 2nd, and ≥3rd), and blastocyst status (expanded, hatching, and hatched). The LBR achieved in high-grade D6 blastocyst transfer was significantly higher than that in low-grade D5 blastocyst transfer (50.43% vs. 40.70%, aOR 1.54 95% CI 1.05–2.26, *p* = 0.027) and is comparable with that in high-grade D5 blastocyst transfer (vs. 57.60%, aOR 0.91, 95% CI 0.62–1.35, *p* = 0.651) (**Table S3**). The ORs of early pregnancy outcomes between four groups are described in **Figure S1**. In addition, to evaluate which factor has a more significant effect on the LBR in the multivariable logistic regression model (**Table S4**), the OR of blastocyst grading (high grade vs. low grade, 1.75, 95% CI 1.53–2.01) was greater than the blastocyst day (D5 vs. D6, 1.40, 95% CI 1.15–1.71).

**Table 3:** Adjusted odds ratio of live birth rate in a multivariate logistic regression analysis.

Blastocyst	Non-adjusted	Model I	Model II
	OR (95% CI), *p*	aOR (95% CI), *p*	aOR (95% CI), *p*
Day 6 high grade vs. Day 5 low grade	**1.48 (1.02, 2.16), 0.041**	**1.54 (1.05, 2.26), 0.027**	**1.48 (1.01, 2.19), 0.047**

AOR, adjusted multivariable logistic regression odds ratio.

Model I adjusted for female age (smooth), parity (0, 1, and ≥2), ET times (1st, 2nd, and ≥3rd), and blastocyst status (expanded, hatching, and hatched).

Model II adjusted for female age (smooth), parity (0, 1, and ≥2), ET times (1st, 2nd, and ≥3rd), blastocyst status (expanded, hatching, and hatched), etiology of infertility, type of infertility, and FBT protocol.

Statistically significant values are highlighted in bold.

### Chromosome aneuploidy analysis

From February 2013 to December 2021, 796 PGT-A consecutive cycles were performed. A total of 2,943 blastocysts were biopsied for aneuploidy analysis (**Table 4** and **Figure 1**). The euploidy rate was highest among high-grade D5 blastocysts (52.84%) and lowest among low-grade D6 blastocysts (41.02%). There were 54.55% of euploid blastocysts (30/55) among high-grade D6 blastocysts, significantly higher than the 41.39% of euploid blastocysts (565/1,365) among low-grade D5 blastocysts (p < 0.001).

**Table 4:** Chromosome aneuploidy analysis according to blastocyst day and morphology grading.

	Day 5 blastocyst		Day 6 blastocyst		*p*-value
	High grade	Low grade	High grade	Low grade	Day 5 low grade vs. Day 6 high grade
N	916	1,365	55	607	
Euploid	484 (52.84%)	565 (41.39%)	30 (54.55%)	249 (41.02%)	**<0.001**
<35 years	404 (44.10%)	466 (34.14%)	19 (34.55%)	208 (34.27%)	**<0.001**
≥35 years	80 (8.73%)	99 (7.25%)	11 (20.00%)	41 (6.75%)	**0.009**
Aneuploid	416 (45.41%)	764 (55.97%)	25 (45.45%)	338 (55.68%)	–
N/A	16 (1.75%)	36 (2.64%)	0	20 (3.29%)	–

Chi-square for categorical variables.

Statistically significant values are highlighted in bold. N/A, Not applicable.

## Discussion

The primary finding of this study is that the high-grade D6 blastocyst transfer achieved a higher chance of live birth than low-grade D5 blastocyst transfer and was comparable to high-grade D5 blastocyst transfer (**Tables S2** and **S3**). Consistent with the current ART practice that all D5 blastocysts are preferred to be transferred compared to D6 blastocysts, our data suggested that high-grade D6 blastocysts are preferred to be transferred first compared with low-grade D5 blastocysts. Meanwhile, the euploidy rate in high-grade D6 blastocysts was also higher than that in low-grade D5 blastocysts from PGT analysis, which also supports this priority of blastocyst selection (high-grade D6 > low-grade D5). In addition, no adverse perinatal outcomes were observed between D5 and D6 blastocysts following frozen-thawed embryo transfer. In view of the results of this study, a fine-tuning of blastocyst selection is proposed in order to shorten the time of conception: D6 blastocysts with high quality are preferred to be transferred compared to D5 blastocysts with low quality when both are available in FBTs.

### D5 vs. D6 blastocysts in pregnancy outcome

Numerous studies have suggested a higher pregnancy outcome from blastocysts developing on D5 than the slower-developing D6 blastocysts (3, 13, 14). One reason is that most of them are based on overall performances. However, results would change when the quality classification of blastocysts (e.g., morphological score) is considered. For example, one meta-analysis reported that there was no difference in clinical pregnancy, ongoing pregnancy, and LBR between D5 and D6 blastocyst transfers with the same morphological quality (15). Kaye reported a similar clinical ongoing pregnancy rate between D5 and D6 blastocysts, regardless of vitrification and slow freezing method (16). Toukhy also reported a comparable LBR between the two groups (D5 vs. D6, 29% vs. 28.5%) when both are high-grade blastocysts (17). Under the control of embryo quality, Yang reported that D6 blastocysts had a similar clinical pregnancy rate (52.4% vs. 52.6%) and implantation rate (38.9% vs. 35.6%) compared to D5 blastocysts when both high-quality embryos were transferred in frozen-thawed cycles (18). In Cimadomo’s study, the LBR after high-grade D6 blastocysts (excellent and good, 28.44%) was even higher than after low-grade D5 blastocysts (average and poor, 22%) (19). Therefore, the high-grade D6 blastocyst can achieve a higher LBR than the low-grade D5 blastocyst, or even a comparable LBR with the high-grade D5 blastocyst.

Indeed, a few conflicting results also exist in the literature. Ferreux reported 18.7% of LBR in D6 blastocysts with high quality, which is lower than 23.5% of LBR in D5 blastocysts with low quality (4). One of the main reasons is that their choice of the embryo to transfer was not based on the day of expansion but exclusively dependent on its quality according to Gardner scoring. This may lead to a selection bias in that a substantial number of low-grade D5 blastocysts missed opportunities for transfer, since the proportion of D6 blastocysts was significantly higher than that in our study (26.21% vs. 13.71%). We agree that the choice of blastocyst selection should be primarily dependent on its quality; however, it seems unreasonable to ignore the day of expansion (D5 vs. D6). The D5 blastocyst is still recommended to be transferred first compared to the D6 blastocyst especially when the morphological scores are similar (e.g., both high grade or low grade).

### D5 vs. D6 blastocysts in PGT analysis

The embryos with a slower development progress were reported to be associated with chromosomal abnormalities (20). The higher risk of aneuploidy in D6 blastocysts could conceivably explain the unfavorable clinical outcomes compared with D5 blastocysts. However, blastocyst morphology was also related to the rate of euploidy, which might be stronger than expansion day (21). In Capalbo’s study (22), the euploidy rate of blastocysts was 56.4% for excellent, 39.1% for good, 42.8% for average, and 25.5% for poor morphology, but the euploidy rate from D5 to D7 was 46.6%, 39.8%, and 43.5%, respectively. In another study, the euploidy rate was found to be 73.2% for blastocysts with good morphology, 50% for average, and 40.5% for poor morphology groups (23), whereas the D5 blastocysts had a euploidy rate of 54.6% vs. 42.8% for D6 blastocysts (2). Although statistical analysis is not available, the variation of euploidy rate in morphology grading (high grade vs. low grade) seems to be greater than that in expansion day (D5 vs. D6) of blastocysts. These data suggested that the poor embryo quality may have a greater impact on the aneuploidy rate than the delayed blastocyst days. Our PGT data illustrated a higher euploidy rate in high-grade D6 blastocysts than in low-grade D5 blastocysts (54.55% vs. 41.39%, **Table 4**), even more obvious among advanced age women (20.00% vs. 7.25%). Even though there was a comparable euploidy rate when compared with low-grade D5 blastocysts (34.14% vs. 34.55%, **Table 4**), high-grade D6 blastocysts have the added advantage for embryo transfer due to a higher LBR. Therefore, the data from chromosome screening support our conclusion that high-grade D6 blastocysts are preferred to be transferred than low-grade D5 blastocysts.

### D5 vs. D6 blastocysts in perinatal outcomes

In view of the slower progression of D6 blastocysts, which may affect perinatal outcomes, we examined the obstetric and neonatal outcomes. In our study, no difference was observed between four groups in terms of preterm birth rate, very preterm birth rate, and live birth weight of newborns. Our results are in line with a propensity score-matched analysis (24), in which the obstetric and neonatal outcomes between D5 and D6 vitrified blastocyst transfers were similar. Some studies indicated that delayed blastocyst expansion (D6 vs. D5) is prone to achieve heavier newborns than D5 blastocysts due to the extended culture *in vitro* (25). However, our data did not support the hypothesis. It is reassuring that no adverse perinatal outcomes were observed between D5 and D6 blastocysts following frozen-thawed embryo transfer.

There is a case in which a small number of blastocysts that have expanded on D5 but vitrified on D6 were mixed in D6 blastocyst transfer. Data from Elgindy suggested a better implantation and pregnancy rates with earlier expanding blastocysts regardless of the time of transfer (26). In the present study, the blastocysts were routinely observed both at 9 a.m. and at 3 p.m. on D5 and vitrified at the expanded stage. Owing to the precautions, this part of blastocysts that expanded on D5 but cryopreserved on D6 is relatively small.

### Strengths and limitations

One strength of the study is that it examines the effect of delayed blastocyst development combined with blastocyst quality. Another strength of this study is its large sample size, including more than 4,000 single-blastocyst transfers. In general, D6 blastocysts selected for transfer are typically cases where all D5 embryos were used up or only D6 blastocysts were available for frozen-thawed embryo transfer, which thereby decreased the chance of embryo transfer for D6 blastocysts. The high order of transfer times in the D6 blastocyst group would increase the proportion of patients with poor prognosis in the D6 group. Despite all this, the pregnancy outcome was still favorable for high-grade D6 blastocysts. The main limitation is that the morphology assessment is subjective and can be operator-dependent. In this study, six embryologists were authorized for blastocyst morphology assessments. This study provided evidence for embryo selection in FET cycles, but not in fresh embryo transfer cycles. Nevertheless, because of the nature of this observational study, the conclusions of this study should be further confirmed by multicenter data as it seems difficult to design a randomized controlled trial.

## Conclusions

In conclusion, high-grade D6 blastocyst transfer achieved a higher LBR than low-grade D5 blastocyst transfer and a comparable LBR to high-grade D5 blastocyst transfer. Meanwhile, a higher euploidy rate was also observed in high-grade D6 blastocysts than in low-grade D5 blastocysts. In addition, no adverse perinatal outcomes were observed between D5 and D6 blastocysts following frozen-thawed embryo transfer. Our data suggested that D6 blastocysts with high quality are preferred to be transferred compared to D5 blastocysts with low quality when both are available for transfer. Consistent with the current ART practice that all D5 blastocysts are preferred to be transferred compared to D6 blastocysts, our data suggested a fine-tuning of blastocyst selection to shorten the time of conception in IVF.

## Data availability statement

The raw data supporting the conclusions of this article will be made available by the authors, without undue reservation.

## Ethics statement

The study was reviewed and approved by the Ethics Committee of Northwest Women’s and Children’s Hospital (No. 2022007). Written informed consent for participation was not required for this study in accordance with the national legislation and the institutional requirements.

## Author contributions

WS designed this study and drafted the manuscript. HZ, XX, and LC contributed to data acquisition, analyses, and data interpretation. JS revised the manuscript. All authors read and approved the final manuscript.
