# Supplementary material for: Live birth rate following frozen-thawed blastocyst transfer is higher in high-grade day 6 blastocysts than in low-grade day 5 blastocysts
Source: Front Endocrinol (Lausanne). 2023 Jan 4;13:1066757. doi: 10.3389/fendo.2022.1066757 (PMC9846233; doi:10.3389/fendo.2022.1066757)
Supplement: Supplementary file 1 [file DataSheet_1.docx]

Supplementary Material

# Supplementary Tables

Table 1 Patient and cycle characteristics according to the blastocyst day and morphology grading between four groups.

|  | Day 5 | | Day 6 | |  |
| --- | --- | --- | --- | --- | --- |
|  | High-grade | Low-grade | High-grade | Low-grade | P-value |
| N | 1835 | 1791 | 115 | 461 |  |
| Female age at OPU | 29.00 (27.00-32.00) | 30.00 (28.00-33.00) | 30.00 (28.25-33.00) | 30.00 (28.00-33.00) | **<0.001** |
| Female age at ET | 29.00 (27.00-32.00) | 31.00 (28.00-34.00) | 31.00 (29.00-34.00) | 31.00 (28.00-34.00) | **<0.001** |
| Female age at ET |  |  |  |  | **<0.001** |
| <35 y | 1613 (87.90%) | 1411 (78.78%) | 89 (77.39%) | 361 (78.31%) |  |
| >=35 y | 222 (12.10%) | 380 (21.22%) | 26 (22.61%) | 100 (21.69%) |  |
| BMI | 21.76 (19.96-24.00) | 22.02 (20.02-24.40) | 22.00 (19.83-23.92) | 21.88 (20.20-24.09) | 0.152 |
| AFC | 15.00 (11.00-20.00) | 12.00 (9.00-16.75) | 12.00 (9.00-16.00) | 11.00 (8.00-16.00) | **<0.001** |
| Etiology of infertility |  |  |  |  | **<0.001** |
| pelvic-tubal factor | 881 (48.01%) | 887 (49.53%) | 66 (57.39%) | 240 (52.06%) |  |
| PCOS | 382 (20.82%) | 224 (12.51%) | 14 (12.17%) | 49 (10.63%) |  |
| male factor | 235 (12.81%) | 192 (10.72%) | 11 (9.57%) | 74 (16.05%) |  |
| other reasons | 337 (18.37%) | 488 (27.25%) | 24 (20.87%) | 98 (21.26%) |  |
| Type of infertility |  |  |  |  | <0.**001** |
| primary | 883 (48.78%) | 677 (38.42%) | 46 (40.71%) | 202 (44.01%) |  |
| secondary | 927 (51.22%) | 1085 (61.58%) | 67 (59.29%) | 257 (55.99%) |  |
| Gravidity |  |  |  |  | <0.001 |
| 0 | 887 (48.34%) | 698 (38.97%) | 46 (40.00%) | 197 (42.73%) |  |
| 1 | 521 (28.39%) | 513 (28.64%) | 33 (28.70%) | 123 (26.68%) |  |
| >=2 | 427 (23.27%) | 580 (32.38%) | 36 (31.30%) | 141 (30.59%) |  |
| Parity |  |  |  |  | **<0.001** |
| 0 | 1472 (80.22%) | 1254 (70.02%) | 82 (71.30%) | 355 (77.01%) |  |
| 1 | 341 (18.58%) | 500 (27.92%) | 31 (26.96%) | 94 (20.39%) |  |
| >=2 | 22 (1.20%) | 37 (2.07%) | 2 (1.74%) | 12 (2.60%) |  |
| ET times |  |  |  |  | **<0.001** |
| 1st | 1355 (73.84%) | 636 (35.51%) | 28 (24.35%) | 58 (12.58%) |  |
| 2nd | 410 (22.34%) | 944 (52.71%) | 65 (56.52%) | 303 (65.73%) |  |
| >=3rd | 70 (3.81%) | 211 (11.78%) | 22 (19.13%) | 100 (21.69%) |  |
| Blastocyst status |  |  |  |  |  |
| expanded | 1822 (99.29%) | 1739 (97.10%) | 106 (92.17%) | 431 (93.49%) | **<0.001** |
| hatching | 10 (0.54%) | 38 (2.12%) | 5 (4.35%) | 14 (3.04%) |  |
| hatched | 3 (0.16%) | 14 (0.78%) | 4 (3.48%) | 16 (3.47%) |  |
| FBT protocol |  |  |  |  | 0.546 |
| natural cycle | 198 (10.80%) | 217 (12.12%) | 14 (12.17%) | 58 (12.61%) |  |
| artificial cycle | 1636 (89.20%) | 1573 (87.88%) | 101 (87.83%) | 402 (87.39%) |  |
| Endometrial thickness | 10.00 (9.20-11.50) | 10.00 (9.00-11.20) | 10.55 (9.03-11.97) | 10.00 (9.20-11.30) | 0.408 |

AFC, antral follicle count；BMI, body mass index; ET, embryo transfer; FBT, frozen-thawed blastocyst transfer; OPU, oocyte pick-up; PCOS，polycystic ovary syndrome.

Median （Q1-Q3） / N (%);

Kruskal Wallis test for continuous variables;

Chi-square for categorical variables;

Statistical significant values are highlighted in bold.

Table 2 Perinatal outcomes according to the blastocyst day and morphology grading.

|  | Day 5 blastocyst | | Day 6 blastocyst | |  |
| --- | --- | --- | --- | --- | --- |
|  | High-grade | Low-grade | High-grade | Low-grade | P-value |
| Biochemical pregnancy | 1363 (74.28%) | 1052 (58.74%) | 78 (67.83%) | 216 (46.85%) | **<0.001** |
| Clinical pregnancy | 1274 (69.43%) | 951 (53.10%) | 72 (62.61%) | 181 (39.26%) | **<0.001** |
| Live birth | 1057 (57.60%) | 729 (40.70%) | 58 (50.43%) | 137 (29.72%) | **<0.001** |
| Preterm deliveries (<37 wk) | 99 (9.37%) | 82 (11.25%) | 8 (13.79%) | 14 (10.22%) | 0.479 |
| Very preterm deliveries (<32 wk) | 15 (1.42%) | 9 (1.23%) | 0 (0.00%) | 0 (0.00%) | 0.428 |
| Birth weight | 3301.73 ± 553.66 | 3315.53 ± 580.63 | 3346.90 ± 422.17 | 3247.40 ± 463.25 | 0.531 |
| <1500g | 11/1079 (1.02%) | 11/740 (1.49%) | 0/58 | 0/146 | - |
| <2500g | 69/1079 (6.39%) | 52/740 (7.03%) | 1/58 (1.72%) | 7/146 (4.79%) | 0.356 |
| >4000g | 104/1079 (9.64%) | 70/740 (9.46%) | 3/58 (5.17%) | 9/146 (6.16%) | 0.385 |
| Birth weight in singlet pregnancy | 3350.00 (3000.00-3650.00) | 3400.00 (3050.00-3700.00) | 3375.00 (3025.00-3700.00) | 3300.00 (3000.00-3530.00) | 0.979 |
| Sex ratio (female/male) | 488/591 | 362/378 | 28/30 | 76/70 | 0.265 |

Median （Q1-Q3） / N (%);

Kruskal Wallis test for continuous variables;

Chi-square for categorical variables;

Statistical significant values are highlighted in bold.

Table 3 Adjusted odds ratio of live birth rate in a multivariate logistic regression analysis.

| Blastocyst | | | Live birth rate | Model I | Model II |
| --- | --- | --- | --- | --- | --- |
|  |  |  |  | aOR (95% CI), P | aOR (95% CI), P |
| Blastocyst | Day 5 | High-grade | 57.60% | Ref | Ref |
|  |  | Low-grade | 40.70% | 0.59 (0.51-0.68), **<0.001** | 0.59 (0.51, 0.68), **<0.001** |
|  | Day 6 | High-grade | 50.43% | 0.91(0.62-1.35), 0.651 | 0.89 (0.60, 1.31), 0.546 |
|  |  | Low-grade | 29.72% | 0.39(0.30-0.49), **<0.001** | 0.38 (0.30, 0.48), **<0.001** |

AOR, adjusted multivariable logistic regression odds ratio.

Model I adjusted for female age (smooth), parity (0,1 and >=2), ET times (1st, 2nd, and >=3rd), and blastocyst status (expanded, hatching, and hatched).

Model II adjusted for female age (smooth), parity (0,1 and >=2), ET times (1st, 2nd, and >=3rd), blastocyst status (expanded, hatching, and hatched), etiology of infertility, type of infertility and FBT protocol.

Statistical significant values are highlighted in bold.

Table S4 The multivariable logistic regression model of live birth

|  | OR | 95% CI | P |
| --- | --- | --- | --- |
| Female age | 0.98 | 0.97- 0.98 | **<0.001** |
| Parity |  |  |  |
| 0 | ref | ref | - |
| 1 | 1.19 | 1.01-1.40 | **0.036** |
| >=2 | 0.75 | 0.44-1.27 | 0.283 |
| ET times |  |  |  |
| 1st | ref | ref | - |
| 2nd | 0.79 | 0.69-0.92 | **0.002** |
| >=3rd | 0.7 | 0.56-0.89 | **0.004** |
| Blastocyst status |  |  |  |
| expanded | ref | ref | - |
| hatching | 1.37 | 0.83-2.25 | 0.216 |
| hatched | 1.2 | 0.61-2.32 | 0.608 |
| Blastocyst grading (high-grade vs low grade) | 1.75 | 1.53, 2.01 | **<0.001** |
| Blastocyst day (day 5 vs day 6) | 1.40 | 1.15-1.71 | **<0.001** |

OR, odds ratio.

Model included female age (≤35 and >35), parity (0,1 and >=2), ET times (1st, 2nd, and >=3rd), blastocyst status (expanded, hatching, and hatched), blastocyst grading (high-grade vs low grade) and blastocyst day (day 5 vs day6).

Statistical significant values are highlighted in bold.

## Supplementary Figures


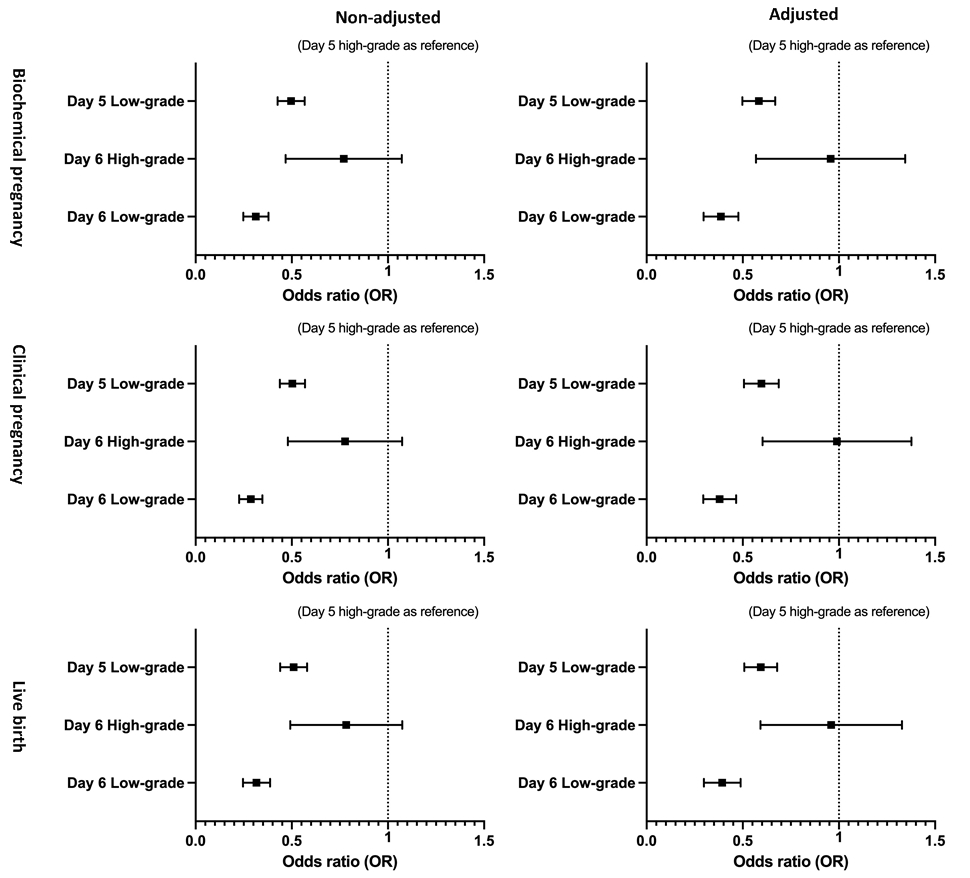


Figure S1 Crude and adjusted odds ratios for pregnancy outcome between high-grade D5 blastocysts (as reference), low-grade D5 blastocysts, high-grade D6 blastocysts and low-grade D6 blastocysts.

OR, odds ratio;

Adjusted for female age (≤35 and >35), parity (0,1 and >=2), ET times (1st, 2nd, and >=3rd), and blastocyst status (expanded, hatching, and hatched).

**
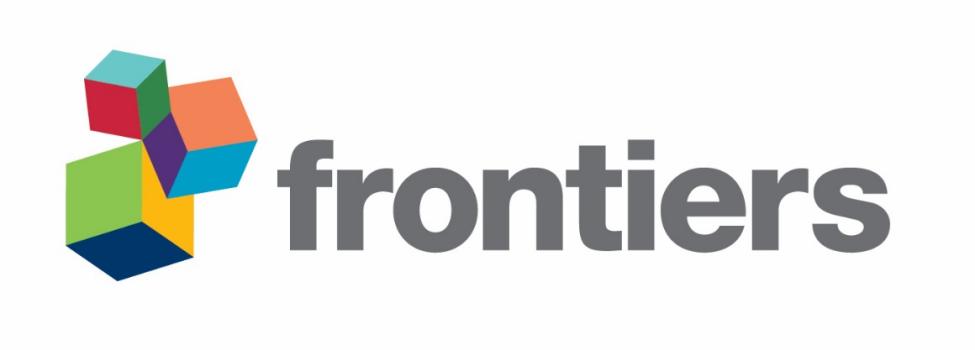
**

**Supplementary Figure 1.** The figure legends are required to have the same font as the main text, 12 point normal Times New Roman, single spaced. Please use a single paragraph for each legend and prepare the figures keeping in mind the PDF layout.
